# Electrochemical Affinity Biosensors Based on Disposable Screen-Printed Electrodes for Detection of Food Allergens

**DOI:** 10.3390/s16111863

**Published:** 2016-11-05

**Authors:** Alina Vasilescu, Gilvanda Nunes, Akhtar Hayat, Usman Latif, Jean-Louis Marty

**Affiliations:** 1 International Centre of Biodynamics, 1B Intrarea Portocalelor, sector 6, 060101 Bucharest, Romania; avasilescu@biodyn.ro; 2 Technological Chemistry Department, Federal University of Maranhão, CCET/UFMA, Av. Portugueses, Cidade Universitária do Canga, 65080-040 São Luis, MA, Brazil; gilvanda.nunes@hotmail.com; 3 Interdisciplinary Research Centre in Biomedical Materials (IRCBM) COMSATS Institute of Information Technology (CIIT), 54000 Lahore, Pakistan; akhtarhayat@ciitlahore.edu.pk (A.H.); usmanlatif@ciitlahore.edu.pk (U.L.); 4 BAE Laboratory, Université de Perpignan Via Domitia, 52 Avenue Paul Alduy, 66860 Perpignan, France

**Keywords:** screen-printed electrode, allergen, biosensor, electrochemical detection, aptamer, antibody

## Abstract

Food allergens are proteins from nuts and tree nuts, fish, shellfish, wheat, soy, eggs or milk which trigger severe adverse reactions in the human body, involving IgE-type antibodies. Sensitive detection of allergens in a large variety of food matrices has become increasingly important considering the emergence of functional foods and new food manufacturing technologies. For example, proteins such as casein from milk or lysozyme and ovalbumin from eggs are sometimes used as fining agents in the wine industry. Nonetheless, allergen detection in processed foods is a challenging endeavor, as allergen proteins are degraded during food processing steps involving heating or fermentation. Detection of food allergens was primarily achieved via Enzyme-Linked Immuno Assay (ELISA) or by chromatographic methods. With the advent of biosensors, electrochemical affinity-based biosensors such as those incorporating antibodies and aptamers as biorecognition elements were also reported in the literature. In this review paper, we highlight the success achieved in the design of electrochemical affinity biosensors based on disposable screen-printed electrodes towards detection of protein allergens. We will discuss the analytical figures of merit for various disposable screen-printed affinity sensors in relation to methodologies employed for immobilization of bioreceptors on transducer surface.

## 1. Introduction 

Food allergies are believed to affect 2%–3% of adults and up to 10% of children in industrialised countries and vast majority of allergies persist through lifetime [1]. Allergens in food are proteins from eight main groups—nuts and tree nuts, fish, shellfish, wheat, soy, eggs and milk—which trigger severe adverse reactions in the human body, involving IgE-type antibodies. Sensitive detection of allergens in a large variety of food matrices and appropriate labelling rules have become increasingly important considering the emergence of functional foods and food manufacturing technologies involving the use of allergen proteins. Raising consumer awareness about new technologies is also important as some industrial uses of allergens proteins are less obvious for the consumers. For example, proteins derived from eggs, milk, wheat or fish gelatin can be used as fining agents in the wine industry [2]. Lysozyme from hen egg is also allowed as an additive (antimicrobial agent) in wine, cheese, sausages, etc. In the EU there are 14 food ingredients that are considered allergens: eggs, milk, peanuts, nuts (almonds, hazelnuts, walnuts, cashews, pecan nuts, Brazil nuts), gluten-containing cereals, lupin, soybeans, celery, mustard, sesame seeds, fish, crustaceans, molluscs and sulphites. The presence of allergens in food has to be declared on its label according to current legislation (e.g., Food Allergen Labeling and Consumer Protection Act of 2004 (FALCPA 2004, Public Law 108–282, Title II in the United States, and the Directive 2000/13/EC, as amended by Directives 2003/89/EC and 2007/68/EC, in the European Union). In Japan, food labelling is mandatory for seven allergens (egg, milk, wheat, buckwheat, peanut, shrimp/prawn and crab) if these are present in food at more than 10 ppm [3]. Undeclared allergens in food and accidental contamination are risk factors, that authorities worldwide aim to manage by various control mechanisms. The number of notifications by the Rapid Alert System for Food and Feed (RASFF) in Europe increased significantly in 2015 compared to previous years and the majority of notifications concerned milk, followed by egg and peanut, soya and gluten [4]. Food Allergy Research and Education (FARE) alerts published in US during 2016 include undeclared milk in organic ranch dressing or pretzel crisps, soy in chicken wrap, pistachio and soy in meat products, peanuts in almond butter, ice cream, oyster crackers or barbeque sauce, milk and soy in potato chips and dietary supplements and hazelnuts in butter cookies [5]. Several examples of 2016 food recall warnings from Canadian Food Inspection Agency [6] include undeclared milk in rice beverages, fully cooked smoked sausages or in coffee mixes, undeclared egg in meal replacement powder or milk, soy, mustard and wheat in cooked ham, to name but a few. These and similar examples worldwide underline the importance of extended analytical control with regards to allergens.

Nonetheless, allergen detection in processed foods is a challenging endeavor [1,7], as allergens are proteins which may be degraded during food processing steps implying heating or fermentation. Detection of food allergens is achieved via methods focused on the biorecognition of the protein itself (most widely used being Enzyme-Linked Immuno Assay–ELISA), methods detecting the DNA encoding allergen proteins (e.g., based on Polymerase Chain Reaction (PCR)) or by chromatographic methods with detection by mass-spectrometry, fluorescence or UV-VIS spectrometry [1]. 

With progress in understanding about the function of living organisms, scientists have sought to apply this knowledge to detect trace amounts of biochemicals in complex systems using bioreceptors from biological organisms. They have developed biosensors as a new mean of analytical and chemical analysis [8]. The promise of biosensors, from the very first work by Clark and Lyons [9] is to provide an alternative to classical methods in medicine, agriculture, food safety, bioprocessing, environmental and industrial monitoring. Because of their exceptional performance capabilities, which include high specificity and sensitivity, rapid response, low cost, relatively compact size and user-friendly operation, biosensors have become an important tool for the detection of a variety of compounds relevant for food quality and safety, including food allergens. Whole cells, enzymes, antibodies, aptamers or molecularly imprinted polymers (MIPs, also called “artificial antibodies”) have been used for this purpose in biosensors in conjunction with different detection modes (optical, electrochemical, mechanical, etc.). For food allergens, choosing the appropriate bioreceptor needs to take into account the changes in protein structure due to food processing or due to sample extraction and preparation protocol used before the actual allergen analysis. Specific bioreceptors for hydrolysed or heated form of proteins and adaptation of sample preparation procedures can be used to overcome these challenges. The reader is referred to excellent recent reviews [7,10] for a detailed illustration of the many challenges associated with allergen detection. Electrochemical biosensors have been shown to provide simple, cost-effective analysis with significant reduction in the time per analysis compared to classic ELISA [10,11,12,13]. Among various types of electrodes in electrochemical biosensors, the flexibility in design allowed by the screen-printing process enables different sensor configurations [14,15], various possibilities for attaching the biorecognition element, to change electrode material by simply changing the ink to be printed, thus allowing to include mediators, nanoparticles, graphene etc. In this review paper, we highlight the success achieved in the design of electrochemical affinity biosensors based on disposable screen-printed electrodes towards detection of protein allergens. We will discuss the analytical figures of merit for various disposable screen-printed affinity sensors in relation to methodologies employed for immobilization of bioreceptors on transducer surface.

## 2. Advantages of Electrochemical Biosensors Based on Screen-Printed Electrodes 

Starting from bulky electrodes and beaker type electrochemical cells and going from mercury electrodes to interfaces based on bismuth and carbon much progress was made in electroanalysis in search of reproducible, inert electrodes, with less background current, and wide potential range [16,17,18]. These electrode materials were very promising in electro-analytical chemistry but lag behind in many features such as portability, low cost, low sample volume, and on-site analysis. Screen printing is a universally adaptive technique which has enabled scientists to eliminate the disadvantages present in bulky electrodes by designing inexpensive, portable, easy-to-use and disposable electrochemical sensors for on-site analysis of various analytes, which are called “screen-printed electrodes” (SPEs) [19,20]. Screen printing technology offers a way to fabricate a whole electrode system consisting of working, auxiliary and reference electrode on a single miniaturized bendable substrate [21]. The best example of commercialized screen printed electrode is the glucose sensor for diabetic patients which is a billion dollar industry [22,23,24] and the demand of this kind of electrochemical sensors based on SPEs for different other analytes will continuously increase in coming years. 

**Figure 1:**
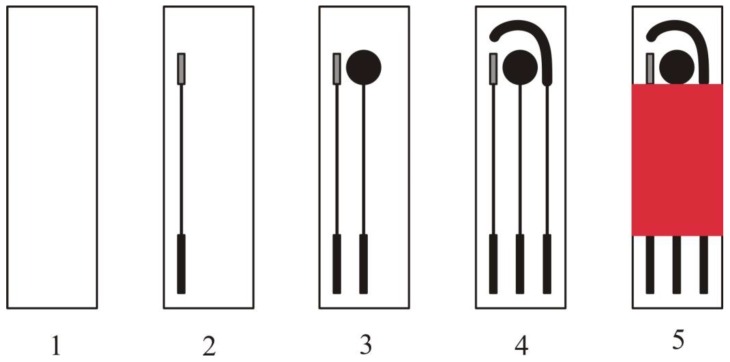
Fabrication of SPEs having three electrode system on chemically inert substrate (**1**). It involves screen printing of reference (**2**), working (**3**), and auxiliary electrode (**4**) on substrate followed by printing with protection paste (**5**).

The fabrication process of SPEs has already been described in a review article by Li et al. [25]. An electrochemical sensor is fabricated by screen printing a three electrode system (working, counter and reference electrode) on a chemically inert substrate as shown in Figure 1. These SPEs work as a transducer and convert a chemical or biological reaction into detectable signals.

The special ink used for screen printing of electrodes is comprised of particles, binders and other additives. Its exact composition is patented and not disclosed by the vendors, any change in the composition of ink affecting the overall performance of SPEs [26,27,28,29]. The performance of these SPEs can be enhanced by using ultrasonic waves which improves mass transport and removes surface active species [30]. Moreover, morphology of electrodes can be altered by varying polymeric binder composition in ink during screen printing [31]. The surface of these SPEs can also be modified in order to use them for particular applications. Currently there are several companies commercialising screen-printed electrodes made of different materials, modified with various types of nanomaterials, mediators or proteins/enzymes: Dropsens (Llanera, Spain), The Gwent Group (Pontypool, UK), BVT Technologies (Brno, Czech Republic), BST Bio Sensor Technology GmbH (Berlin, Germany), Rusens Ltd (Moscow, Russia), etc. To these add the efforts made by various research groups to enrich the portfolio of low cost, performant biosensors by taking advantage of screen-printing equipment available in their research labs. Among representative examples of biosensors with direct application to food analysis are those developed by some of us with screen-printed electrodes fabricated at University of Perpignan, France for the detection of D-lactic acid, acetaldehyde, okadaic acid, ochratoxin A and lysozyme [15,32,33,34].

The electrode materials used in screen-printed electrochemical transducers for allergen detection were C, Pt and Au. Gold electrodes were preferred in genosensors, as they afford easy immobilisation of thiolated DNA capture probes. The majority of screen-printed biosensors for allergens were carbon-based and their modification and functionalization aimed to promoting faster charge transfer, stable attachment of bioreceptors and enhancing the loading capacity with biomolecules. Available strategies for introducing functional groups at electrode surface for bioreceptor attachment include: treatment with an oxygen plasma [35] or pre-anodization [36] to promote the formation of carboxylic groups on the surface and modification with a diazonium salt formed in situ. The performance of pre-anodized SPEs was shown to be much better in terms of reduction in overpotential and oxidation peaks than oxygen plasma treated SPEs. Electrochemical reduction of a diazonium salt on electrode surface leads to the formation of an aryl centered radical. The formed aryl diazonium binds covalently to the surface by the spontaneous release of nitrogen. Diazonium salts modified with various functional groups have been used for immobilization of proteins, antibodies and other molecules [37,38]. For allergen detection in particular electrodeposition of diazonium salts served to introduce either carboxylic [34,39], or amine groups [40] at electrode surface. Screen-printed carbon electrodes (SPCE) were also modified with nanomaterials such as graphene [40,41], carbon nanotubes [42] and Au nanoparticles (AuNPs) [43] to facilitate the immobilisation of high amounts of bioreceptor and improve the conductivity of the electrodes. CNTs show enhanced electro-catalytic properties towards different analytes due to the presence of defect/edge like sites on their surface [44,45]. Many types of modified screen-printed electrodes are available commercially and new ones are continuously developed, therefore it is anticipated that new transducers and configurations will be integrated in the future in biosensors for allergen analysis. Moreover, many of the biosensor construction steps discussed in Section 3, currently performed manually, are compatible with mass-production. As their potential will be proven by researchers, various biofunctionalised electrodes are expected to become commercially available as well.

## 3. Design of Sensitive Biosensors for Allergen Based on Screen-Printed Electrodes 

Most of the biosensor literature concerning food allergens detection reports on antibody-based assays, as aptamers have been selected so far only for lysozyme, Ara h 1, gliadin and conglutinin (lupin) allergen. Genosensors relying on DNA probes to capture and detect gene fragments encoding the allergen proteins have also been developed. Not in the least, “artificial antibodies”—molecularly imprinted polymers (MIPs) have been explored as well for allergen detection [46]. MIPs are synthetic polymers able to bind with good affinity a target analyte and were proven to have a great potential to assist with separation and purification of various analytes from real samples. 

The next section of the review paper will focus on the assays formats, immobilization methodologies and transduction approaches employed in the fabrication of screen-printed affinity based biosensors for the evaluation of food allergens.

### 3.1. Bioreceptor Immobilisation and Detection Format

The majority of the biosensors for food allergens reported until now are based on the principle of protein biorecognition with antibodies as bioreceptor element [7]. To these add several aptasensors and genosensors. In aptasensors, allergen recognition is achieved by affinity binding to aptamers, while genosensors are aimed at detecting the genes encoding the allergen, via hybridization with complementary DNA sequences. The recognition abilities of MIPs are due to the complementary in shape and size of the target compounds with cavities in MIPs. These cavities are formed during MIP production by polymerisation in the presence of target analyte. Immunosensor design relies on immobilization of antibody or antigen (allergenic proteins) and monitoring of the affinity binding between antigen and antibody with different types of transducers. The assay formats used with immunosensors are similar to those described for classical immunoassays [47,48,49,50]. Monoclonal and polyclonal antibodies have been successfully integrated with biosensor technology. Polyclonal antibodies are the product of immunization of a living host organism, usually a mammal such as rabbits, goats or sheep. Polyclonal antibodies are synthesized by multiple plasma cells. The major drawback with the use of polyclonal antibodies is the highly heterogeneous nature of the polyclonal antisera. As an alternative, monoclonal antibodies (Mabs) are obtained by growing hybridoma cell lines in culture and are produced in murine or mouse hosts, being very specific for a particular epitope of a food allergen. Therefore, monoclonal antibodies can be considered advantageous over polyclonal antibodies with their less non-specific binding to the food extracts and reduced cross reactivity with food matrix or ingredients. Nonetheless, their production is very laborious and expensive process, and only a limited number of immunosensors based on the monoclonal antibodies are reported in the literature for the detection of food allergens [47,48,49,50]. In the same context, the immobilization of the antibodies/antigen onto a transducer or a support matrix is a crucial step in optimizing the analytical performance of an immunosensor in terms of response, sensitivity, stability and reusability. Physical adsorption is the easiest method to immobilize antibodies and involves van der Walls forces and electrostatic interactions between the Ab/Ag and the transducer surface. The main advantages of this technique are its rapidity and simplicity, while its limitations are random orientation, weak attachment [51], denaturation of immobilized molecules and reduced long term stability of the designed sensor surface. Antibodies/antigens (Ab/Ag) can also be attached covalently to the transducer surface through the formation of a stable bond between the functional groups of Ab/Ag and the transducer support, leading to increased stability of the Ab/Ag. Antibodies or antigens can be stably attached through their amine end groups to carboxyl-functionalised electrodes via carbodiimide chemistry (amine coupling) or to amine-functionalised interfaces via cross-linking with glutaraldehyde. For example, the anti-β-lactoglobulin antibody and an anti-ovalbumin antibody were covalently affixed via glutaraldehyde on 4-aminophenyl, graphene-modified carbon electrodes, to yield β-lactoglobulin [40] and ovalbumin [41] immunosensors, respectively. Immobilisation of β-casein to carboxyl groups on oxygen-plasma treated carbon electrodes, in order to build a competitive biosensor was accomplished via classic carbodiimide chemistry. By the same method, other researchers attached covalently protein A to an electrochemically pretreated C electrode. Furthermore, the β-casein antibody was immobilised on the electrode by affinity for Protein A [36]. Not in the least, another type of strong affinity interaction—biotin/strept(avidin)—can be used for simple and easy immobilization of the Ab/Ag on the surface of nanoparticles [32].

### 3.2. Aptasensors

The nature of antibody production process, leading to batch-to-batch variability, as well as their short shelf life limits their wider application in the analysis of real samples. A new class of molecules, named aptamers hold significant advantages over antibodies and appear as promising recognition tools for analytical applications [52]. Aptamers are short single stranded oligonucleotides, either DNA or RNA that fold into well-defined 3D structures and bind to their ligand by complementary shape interactions, with antibody-like binding ability. They are typically obtained through an in vitro selection procedure, called Systematic Evolution of Ligands by Exponential enrichment (SELEX), first reported in 1990 [53,54]. As they are chemically synthesized, their production does not require animal use and is therefore less expensive and tedious. Aptamers can also be easily labeled with a wide range of reporter molecules such as fluorescent dyes, enzymes, biotin, or aminated compounds, enabling the design of a variety of detection methods [53]. Furthermore, the function of immobilized aptamers can be easily regenerated and aptamers can be reused. Due to these advantages, aptamers can thus be considered as a valid alternative to antibodies or other bio-receptors, in developing various analytical techniques. RNA and DNA aptamers have been selected and employed for the detection of egg lysozyme [53,55,56,57]. Nadal et al. have recently selected an aptamer against the food allergen Lup an1 (from lupine) [58]. In the same context, Tran et al. have successfully designed specific aptamers strands against food allergens including lysozyme [56] and Ara h 1 [59]. Several aptamer sequences for gluten were selected by two research groups, by taking as analytical target either the intact gliadin protein [60] or the immunodominant toxic 33-mer peptide [61]. Despite of the progress in the field of aptasensing, very few reports are available in literature for aptasensing detection of food allergens. However it is an effervescent area of research according to ongoing projects by several research groups. 

Similar to antibodies in immunosensors, aptamer immobilization on transducer`s surface is a crucial step in the development of aptasensors. There are a number of chemical methods for the immobilization of aptamers that are all based on the methods previously developed for single or double strand DNA in genosensors or biosensors for detection of DNA [62]. Aptamers can be immobilized on electrode surface via their 5’-end or the 3’-end, and the development of aptasensors using both positions have been reported [63]. Several studies emphasized that the 3’ end is more suitable because it would simultaneously confer resistance to nuclease after its binding to electrode surface. Physical adsorption by means of electrostatic forces is the simplest strategy to immobilize the aptamer on the transducer surface. This method, however, has the disadvantage of low stability caused by desorption from the surface. The commonly used immobilization techniques to obtain a stable and active aptamer-functionalized surface include covalent coupling [39,42] click chemistry [43] and avidin-biotin affinity [61]. Chemisorption of the thiolated aptamer onto a gold electrode surface [52] could be envisaged as well, similarly to aptasensors for other analytes relevant for food safety. Based on the large size of food allergenic proteins, different assay formats are used in the construction of affinity based biosensors.

### 3.3. Genosensors

Genosensors are aimed to specific recognition of DNA fragments of the genes encoding for the toxic allergen proteins or peptides and rely on the immobilisation on electrode surface of “capture” DNA probes able to hybridize with the target DNA. There is no direct quantitative relation between the quantity of genes encoding the allergen and the amount of allergen in food. However, DNA-based methods including genosensors are very useful and complement protein-targeting methods to identify the presence of allergens in a food matrix. Due to the easiness of the process and knowledge available from microarray development, sensing layers in genosensors are typically obtained by self-assembly of thiolated capture probes on Au electrodes [64,65,66]. 

### 3.4. Sensors Based on MIPS 

Although MIPs have been particularly useful for sample clean up and separations targeting mainly small molecules, protein recognition with MIPs registered important progress in the last years [67]. With regards to allergens, MIPs for lysozyme [68], β-lactoglobulin [69], ovalbumin [70] and peanut protein [71] have been reported. However, with the exception of an older report on lysozyme [46], the potential of MIPs in conjunction with screen-printed electrodes for allergen detections remains untapped.

### 3.5. Assay Formats in Affinity Based Biosensors

Affinity biosensors for the detection of food allergens employed different assay formats, from which most frequently used were sandwich and competitive assays, similarly to ELISA tests. The selection of the format depends on the available reagents and dynamic range required for the particular assay. Sandwich assays allow sensitive and specific detection of target analytes and are appropriate for intact allergen proteins. Allergen fragmentation during food processing (e.g., in the case of gluten in hydrolysed food), results in small peptide fragments which cannot accommodate two binding epitopes. In this case, competitive tests are appropriate, leading to sensitive detection as well. Direct detection was more seldom used; its simplicity and reduction of analysis time due to smaller number of steps could be advantageous for practical applications and should be investigated as well. Antibody and aptamer-based assays commonly share the below discussed formats, while gensensors were all based on a sandwich configuration.

#### 3.5.1. Sandwich Format

A sandwich immunosensor is based on two bioreceptor molecules, which bind to different sites on the bioreceptor or ligand. The highly specific bioreceptor (aptamer/primary antibody) is immobilized to a transducer surface. The antigen is then incubated followed by addition of a secondary labeled detection bioreceptor molecule which can be an antibody or an aptamer. The antigen is “sandwiched” between the two bioreceptor molecules as indicated from the name of the format. The bioreceptor binding affinity to the antigen is a crucial step for the determinant of assay sensitivity. With increase in the antigen concentration, the amount of detection bioreceptor molecule increases resulting in to a higher output signal. The generated response of the sandwich assay is used to determine the amount of target antigen present in the sample [34,59]. 

**Figure 2:**
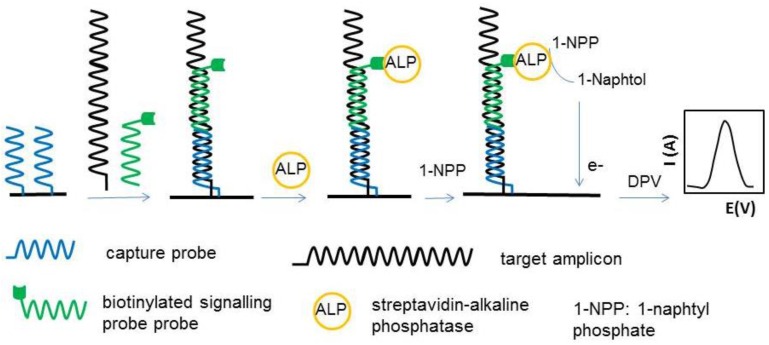
Construction and principle of individual genosensors included in a 8 electrode array for the detecting the presence of hazelnut allergen Cor a 1 in foods as developped by Betazzi et al. [64]. Each electrode was modified with one kind of capture probe. The capture probes included in the array were complementary DNA sequences to several fragments of the genes encoding the expression of hazelnut isoallergens Cor a 1.03 and Cor a 1.04.

In genosensors, the target DNA is hybridized with a capture DNA probe fixed on electrode surface and with a signaling DNA probe. Electrochemical genosensors used in food safety assessment have been recently reviewed [72] and the critical factors determining the analytical performances of genosensors have been discussed in several papers [65,73,74]. The main points to consider in the design of genosensors are: optimal composition of the sensing layer including the density of capture probe, concentration and nature of diluent thiol, the geometry of the capture probe (hairpin or linear), the length of target DNA and the relative position of the recognition part of target DNA, hybridising with target and signalling probes. Lopez et al. [65] focused on optimisation of the sensing phase using Design of Experiments, while Bezazzi et al. [64] studied in more detail the configuration of capture DNA, target DNA and signalling probe with respect to length and position of the signalling probe on the hybridized construct. Optimum results were found when the signalling probe and capture probe were contiguous along the target DNA when hybridised (Figure 2). The main construction steps and the principle of individual genosensors included in the array are illustrated in Figure 2. 

#### 3.5.2. Competitive Binding Format

In case of competitive formats, only one bioreceptor molecule is used to avoid the steric hindrance that occurs if two bioreceptor bind to target analyte. The basic principle of the assays is the immobilization of biomolecules (aptamer/antibody or antigen) onto a support, followed by a competitive process, between free antigen and labeled biomolecules. A fixed amount of labeled ligand and a variable amount of unlabeled ligand is used for detection purpose. As the concentration of the unlabeled ligand is increased, the measured response decreases. There are two types of competitive formats. In the direct competitive immunoassay format, the immobilization surface is coated with bioreceptor molecule. After the fixation step, the competition is allowed to proceed between free antigen from sample and labeled antigen [49]. In the indirect competitive immunoassay format, antigen or antigen conjugates are immobilized onto the transducer platform. The competition is allowed to proceed, by adding free antigen from sample and bioreceptor. Secondary labelled antibody is added in case of immunoassays [37], while labelled aptamer is used in case of aptamer based assay [75].

#### 3.5.3. Non-Competitive Format

Non-competitive format is only used in case of label-free detection. The principle for non- competitive format is the immobilization of the bioreceptor molecule on a transducer surface. The antigen molecules in the sample are incubated directly on the transducer surface. The formation of bioreceptor-antigen complex leads to change the electrical or physical properties of the surface. This change in the behaviour of the surface is directly proportional to the antigen-bioreceptor complex, which is related to the concentration of antigen in the sample [39,76].

#### 3.5.4. Additional Formats in Aptasensing Designs

##### Target-Induced Structure Switching Format (TISS)

In such type of detection methods, the immobilized aptamer undergoes conformational changes upon binding to the target molecules. This change in specific pattern of aptamer leads to the changes of detectable characters, including: (a) the change in position, quantity or status of the covalently attached or adsorbed signal moieties; (b) the change in size or weight of aptamers due to the complex formation between target-aptamer; (c) change in the other properties of the aptamers, such as the ability to stabilize gold nanoparticles. Each of the changes described above induce a change of signal in the aptasensor detection. This mode of detection is suitable for both embedded group and outside-binding group. Upon the formation of aptamer-target complex on the electrode surface, aptamers turn into rigid tertiary structures, such as G-quadruplex, from random conformations. Such conformational changes would induce change in the relative position of signal moieties, leading to change in electrochemical signal [18,33].

##### Target-Induced Dissociation/Displacement Format (TID)

In contrast to target-induced structure switching detection method, dissociation or displacement detection does not rely on the special conformation of the aptamer or the specific target-aptamer complex structures. In this mode, the complementary sequence of aptamer is used as anchor to immobilize the aptamer. After binding to target molecule, the target-aptamer complex is liberated into the solution, leading to detectable signals. Target-induced dissociation/displacement methods are further classified into “signal-off” mode [77], “signal-on” mode [78] and “label-free” mode [79].

**Figure 3:**
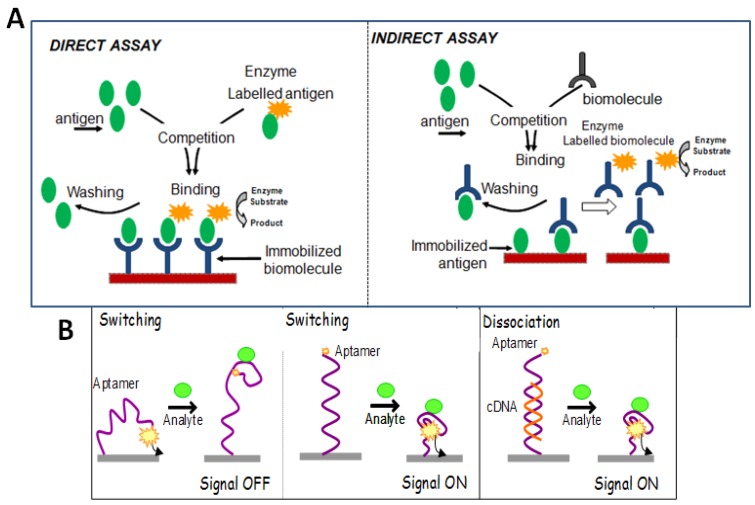
(**A**) Direct and Indirect competitive formats in the affinity based biosensors; (**B**) Additional formats in case of aptamer based assays.

In case of signal-off strategy, the target concentration is determined from the signal suppression. In the absence of target, redox-probe induce electrochemical signal, while after the addition of target, the aptamer-target complex release into solution from immobilization surface, and the electrochemical signal reduces. In construction of signal-on aptasensor mode, the aptamer, hybridized with its complementary sequence is immobilized on the transducer surface. As the target is added, the aptamer sequence is released from double strand to form target-aptamer complex. The remaining complementary sequence is hybridized with another labelled ssDNA, resulting in the production of electrochemical signal (Figure 3A). Further, in case of label free strategy, the release of aptamer due to the formation of target-aptamer complex decreases the interfacial electron transfer resistance, which is used to determine the target molecule concentration (Figure 3). Such a label-free TID strategy was proposed for lysozyme assay [79]. Figure 3 provides an overview and detection principle of the various formats employed in the construction of affinity based biosensors.

### 3.6. Assays Coupling Ligand-Modified Magnetic Beads with Electrochemical Detection with SPEs 

**Figure 4:**
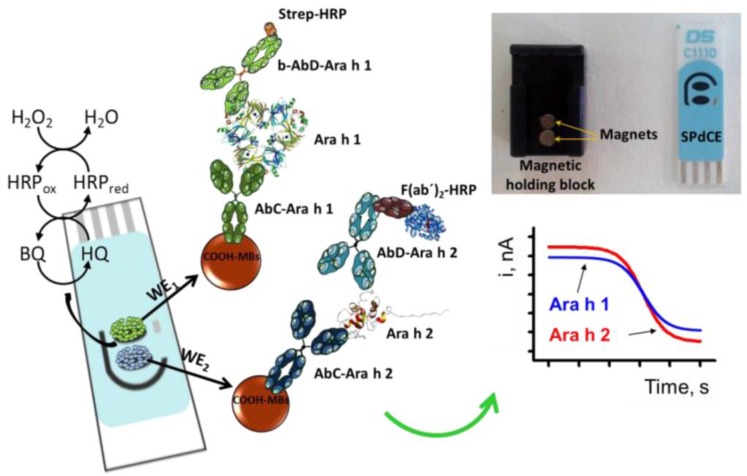
Principle of the simultaneous determination of Ara h 1 and Ara h 2 using screen-printed dual carbon electrodes and amperometric detection. A real picture of the SPdCE and the homemade magnetic holding block is also shown. Reprinted from [90] under Creative Commons Attribution (CC-BY) license (http://creativecommons.org/licenses/by/4.0/).

Among various types of nanomaterials that were applied to increase the analytical performance of electrochemical sensors [80] and bioassays, magnetic nanoparticles have been largely preferred for functionalization of electrode surface because of their high surface to volume ratio [75,81], which facilitates quantitative immobilization [32,82,83,84,85,86]. Commercially available magnetic beads (MBs) modified with carboxyl functionalities, streptavidin, protein A or protein G allow different strategies for easy and stable attachment of biomolecules with preservation of their activity and affinity. Modified MBs have also wide applications in analytical separation and concentration of target analytes from complex real samples. Actuation under a magnetic field to concentrate MBs on an electrode surface for performing affinity recognition followed by regeneration of the original surface for the next assay by switching off the magnetic field offer an easy way to improve the specificity and sensitivity of assays and made from electrochemical magnetoimmunoassays a preferred approach for many researchers. Magnetic nanoparticles actuated in magnetic fields can also be used to transport biomolecules at target sites in microfluidics, while selecting an appropriate frequency of actuation can help improve the signal-to-noise ratio of the assays. Overall, magnetic beads proved to be versatile tools for electrochemical biosensing [82,87,88]. Association of screen-printed electrodes for cost-effective, easy detection with magnetic beads functionalized with high affinity, specific antibodies or aptamers delivered an advantageous approach for the detection of various analytes [88], including food allergens such as ovalbumin [82], β-lactoglobulin [89] Ara h 1 and Ara h 2 [90] and gluten [61]. An illustrative example of the potential and advantages of magnetoimmunosensors was provided by Ruiz-Montiel et al. [90] for the simultaneous detection of Ara h 1 and Ara h 2 using dual screen-printed electrodes. Sandwich immunoassays were performed onto magnetic beads (MBs), immobilised on electrode surface by positioning a magnet under each electrode. The magnetic beads were modified with antibodies specific for Ara h 1 and Ara h 2, respectively. The allergen proteins were captured by the antibodies and sandwiched between them and detection antibodies added in the solution. To translate allergen binding onto the MBs into an electrochemical signal, the detection antibodies were labelled with horseradish peroxidase (HRP), hydroquinone was added as enzyme substrate and the enzymatic product, benzoquinone was electrochemically detected at the surface of screen-printed electrodes as depicted in Figure 4.

Besides simultaneous detection of multiple allergens, the above study emphasized some additional advantages of magnetoimmunoassays, namely shorter analysis time and higher sensitivity compared to ELISA using the same immunoreagents. Thus, the time per assay was reduced from 4 h to 2 h and the detection limits achieved with the biosensor were 18.0 and 0.07 ng·mL^−1^ for Ara h 1 and Ara h 2 compared to 31.5 and 2 ng·mL^−1^ for Ara h 1 and Ara h 2 in ELISA.

The reproducibility of magnetoimmunoassays with electrochemical detection can be improved by manipulating the external magnetic field for uniform dispersion of magnetic beads and enhance the relative motion of beads with respect to surrounding fluid for improved exposure to the analyte irrespective of microfluidic or biological processes.

### 3.7. Other Tests Coupling Affinity Recognition of Allergens with Electrochemical Detection on SPEs

The advantages of SPEs for providing sensitive, low volume, easy and cost-effective detection have been exploited also in bioassays where the biorecognition event took place far from electrode surface. Recently, a lab-on-a-membrane device was described for simultaneous biosensing of casein and gamma−globulin G in milk [91] using Quantum Dot (QD)-labelled antibodies immobilized on two membranes. The membranes were folded and brought on top of screen-printed graphite electrodes. Subsequent acidic dissolution of QDs and detection by ASV allowed for cost effective determination of bovine casein and gamma−globulin G in milk.

### 3.8. Electrochemical Detection Method

The interaction of antigen with bioreceptor molecules at electrochemical transducer surface results in physicochemical changes such as alteration in the surface thickness and change in redox properties. Electrochemical detection methodologies are based on the principle that electroactive species are oxidized or reduced on a working electrode surface under predefined fixed or varying potential. The generated electron flux is measured in the form of output signal in the form of various electrogenerated signals such as voltammetry, electrochemical impedance spectroscopy and amperometry. The generated electrochemical signal is highly dependent on the nature of working electrode material and consequently various types of electrode materials such as doped or undoped carbon, gold, nickel, silver and copper are reported in the literature. These materials offer the advantage of chemical modification in diverse ways to enhance the analytical performance of the biosensors in terms of sensitivity, selectivity and stability. Moreover, biosensors based on electrochemical output signal also offer the opportunity of miniaturization with possibility to design field portable devices [92]. 

Electrochemical impedance spectroscopy is a widely used method to monitor the interfacial electron transfer resistance at the functionalized transducer surfaces. The method presumes the application of a fixed electrical potential, over which is superimposed a small alternative signal. The biosensor is matched to an equivalent electrical circuit and the electron transfer resistance at the interface is measured in the presence of a redox active probe (typically ferricyanide/ferrocyanide). The binding of target allergen to its bioreceptor alters the electron transfer resistance at the electrode solution interface in direct relation with the amount of allergen in solution. Consequently, the electron transfer resistance is most often the parameter used to draw a calibration curve. For example, impedimetric immunosensors for the monitoring of Ara h 1 and Ara h 2 with remarkable analytical figures of merits have been reported in the literature [76,93] based on solid electrodes and similar concepts could be envisaged with screen-printed electrodes as well. Similarly, our group has compared the performance of two aptamers sequences in the detection of lysozyme based on the impedimetric detection with screen-printed aptasensors [39]. 

Voltammetry is another electrochemical technique preferred in biosensing, which involves varying the electrical potential between the working and reference electrodes in a certain range and measurement of the intensity of electrical current due to electrochemical oxidation (or reduction) occurring at electrode surface. Cyclic Voltammetry, Linear Sweep Voltammetry (LSV), Differential Pulse Voltammetry (DPV) and Square Wave Voltammetry are various types of voltammetry methods used in electrochemical biosensors. The intensity of the current associated with the oxidation of a reporter probe added in solution, such as ferrocyanide changes as result of the binding event, due to steric hindrances and/ or electrostatic interactions prompted by the formation of allergen-bioreceptor complex. Changes in current intensity are linked to the amount of allergen in the sample. For example, this principle was used in a label free immunosensor for the detection of β-lactoglobulin [40], where the current due to oxidation of ferrocyanide added in solution decreased upon incubation of the immunosensor with the target and formation of the bulky antibody-antigen complex. In the case of aptamers, one of the simplest schemes used for electrical biosensing measured the current due to hexamine ruthenium, which binds electrostatically to phosphate groups of DNA bases in the aptamers. Aptamer-target binding results in a decrease in the number of freely accessible phosphate groups that could be “titrated” by hexamine ruthenium, hence a decrease in electrochemical signal. 

In the same context, enzyme labels embedded in signalling probes with immunosensors, aptasensors and genosensor assays are widely used to generate electroactive products, whose electrochemical signal can be directly related to the concentration of allergen. Like in ELISA, horseradish peroxidase (HRP) and alkaline phosphatase (ALP) are the enzyme labels used in DNA signalling probes or in secondary antibodies. Alkaline phosphatase catalyzes the dephosphorylation of different substrates. The transformations used with electrochemical screen-printed biosensors for allergens and ALP include, for example the conversion of 1-naphtyl phosphate to 1-naphtol used in a genosensor for hazelnut allergens [64] and in a sandwich biosensor for lysozyme [34]. This step is followed by the oxidation of 1-naphtol by DPV and correlation of the intensity of oxidation current with the quantity of genes encoding hazelnut allergens or, in the second example with the amount of lysozyme in the sample. Another example of transformation catalysed by ALP used in biosensors for allergens is the enzymatic deposition of silver by the reaction between 3-indoxyl phosphate and silver nitrate. This reaction, used in an immunosensor for the detection of Ara h 1 peanut allergen, was combined with electrochemical stripping of silver by LSW to correlate the electrochemical output signal with the concentration of allergen [94].

Other strategies for sensitive voltammetric analysis of allergens involve QDs-labelled bioreceptors [91,95]. These have the advantage that allow simultaneous detection of several protein targets, using specific bioreceptors labelled with different QDs. After the biorecognition event, acidic dissolution of quantum dots labels followed by adsorptive stripping voltammetry provides a very sensitive way for protein detection, the main limitation in simultaneous detection of a high number of proteins being the resolution of voltammetric signals of QDs.

Besides electrochemical impedance spectroscopy and voltammetry, amperometry is the preferred electrochemical detection method in affinity biosensors, including those for allergens. Amperometry relies on measuring the intensity of electrical current produced as result of electrochemical oxidation/reduction of an active compound upon application of a suitable potential between the working electrode (the biosensor) and a reference electrode. As the necessary equipment is simpler compared to both EIS and voltammetry, the combination of amperometric detection with screen-printed electrodes is particularly suitable for simple, robust applications of high commercial potential, as demonstrated by the well-known example of glucose biosensors available in drugstores.

In detection schemes of biosensors for allergens, amperometric detection has been performed in conjunction with biocatalysis by HRP, another largely used enzymatic label besides ALP. As per these strategies, HRP catalyses reactions such as (i) conversion of hydroquinone, in the presence of H_2_O_2_ to benzoquinone (BQ) or (ii) oxidation of ,3,3’,5,5’-tetramethyl benzidine (TMB) in the presence of H_2_O_2_. Reduction of BQ [35,96] and detection of enzymatically oxidised TMB [61,66,97] by amperometry were linked to the amount of allergen present in the sample. 

In addition to enzyme-based amplification, recently reported signal amplification schemes using nanomaterial labels with enzyme-like activity (nanoceria) or with intrinsic electrochemical activity (graphene) could be adopted in the future for allergen biosensing as well.

## 4. Applications of Biosensors Based on Screen-Printed Electrodes for Allergen Detection 

### 4.1. Examples of SPE-Based Biosensors for Allergen Detection

Biosensors for allergens, as emphasized in several reviews [7,10,11,98], rely mainly on antibodies as biorecognition element, as aptamers have been selected so far only for lysozyme, Ara h 1 peanut protein, gliadin (gluten) and β -conglutin lupin protein. However efforts are under way to develop aptamers for other toxic proteins (e.g., hazelnut allergens). Combination of stable, high-affinity bioreceptors with disposable, cost-effective screen-printed electrodes appears as a success recipe that could lead to new commercial analytical tools to complement or partially replace ELISA kits. To ensure practical application, biosensor performances should be evaluated not only in terms of sensitivity and reproducibility, easiness of the test, time per assay and cost of production but also in response to challenges with real samples. Examples of biosensors based on screen-printed electrodes for the detection of allergens given in Table 1 offer only a glimpse of the wide possibilities that could be considered in the future. Many of the biosensing strategies reported in literature for allergens, using different electrode materials [7,10,11,98,99] could be adapted to detection with screen-printed electrodes. Several electrochemical biosensors for the determination of allergens in food samples will be detailed further.

#### 4.1.1. Biosensors for Milk Allergens

Milk is a common food allergen and the group with the highest risk of milk allergy is represented by infants. Undeclared milk on food products’ label was linked, in 2015, to the largest number of allergen alert notifications in Europe recorded by RASFF. The major toxic proteins in milk are caseins, β-lactoglobulin and α-lactalbumin. 

**Figure 5:**
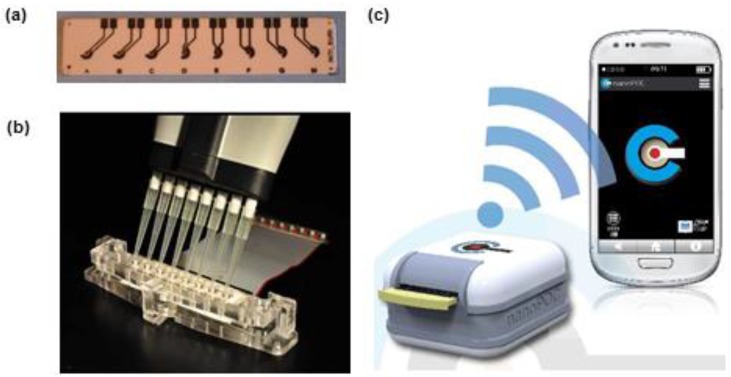
Analytical setup for casein detection including screen-printed electrodes (**a**), set of 8 electrochemical cells (**b**) and portable potentiostat controlled by smartphone via Bluetooth (**c**). Reproduced from [35] under the terms and conditions of the Creative Commons Attribution license (http://creativecommons.org/licenses/by/4.0/).

Caseins are phosphoproteins (αS1, αS2, β, κ), with a molecular weight of 19–25.2 kDa and an isoelectric point of 4.1–5.8 that amount to 80% of the proteins in cow’s milk. Besides being a food ingredient, casein has various industrial applications in paints, plastics, medicine and dentistry, as a fining agent in wines, drug carrier for pharmaceuticals etc. Residual levels of casein in wines are typically very low and therefore not posing threats to consumers with milk allergy [101], as found by a 2012 study focussing on Italian wines fined with potassium caseinate. However the analytical control of casein in food and beverages is important and biosensors have contributed to the variety of sensing strategies available for casein detection. While no aptamer is available yet for casein and specific genosensors still await development, two immunosensors were developed based on screen-printed electrodes. One amperometric biosensor allowing casein detection in the 0–10 ppm range was used in connection with a small, portable potentiostat controlled by a smartphone via Bluetooth communication [35] (Figure 5).

**Figure 6:**
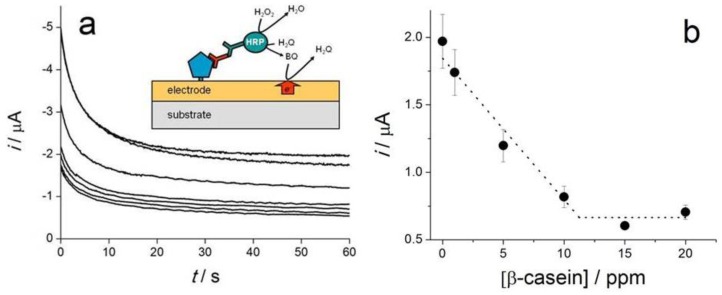
Chronoamperometric detection of benzoquinone formed as result of casein binding in the immunosensor for several casein concentrations (**a**) and calibration curve obtained based on the intensity of the current recorded after 60 s from applying the potential (**b**). Reproduced from [35] by the terms and conditions of the Creative Commons Attribution license (http://creativecommons.org/ licenses/by/4.0/).

The biosensing principle relies on the competition between casein immobilised on the electrode and that contained in the sample, for anti-casein antibodies added in the sample solution. Translation of casein-antibody binding event into an electrochemical signal that is quantitatively correlated with the amount of casein in the sample was achieved with a secondary antibody labeled with HRP and chronoamperometric detection of benzoquinone formed in the enzymatic reaction. The intensity of the current recorded 60 s after applying the potential was taken as analytical signal used to build the calibration curve (Figure 6a,b).

This casein immunosensor illustrates two of the current trends and opportunities available with electrochemical screen-printed biosensors: (i) simple and portable instrumentation, controlled by smartphone and (ii) multiple simultaneous analysis enabled by integration of eight electrochemical cells instead of a single one. This could serve either to increase sample throughput for casein analysis or, by modifying the electrodes with different bioreceptors, can be used to achieve simultaneous detection of multiple analytical targets. 

Considered the most toxic allergen in milk, β-lactoglobulin makes for approximately 10% of milk proteins. It is a 18.4 kDa protein, with an isoelectric point of 5.1, which is dimeric at physiological pH and monomeric below pH 3. Bioanalytical methods developed so far for β-lactoglobulin include a magnetoimmunoassay [89] and an immunosensor relying on screen-printed electrodes [40] (Table 1). Commercial graphene-modified carbon electrodes were functionalised with diazonium salt formed in situ to introduce amine groups at electrode surface [40].

Anti-β-lactoglobulin antibodies were covalently anchored to these groups via glutaraldehyde. Simple, direct detection of β-lactoglobulin was accomplished by DPV, by measuring the decrease in current due to electrochemical oxidation of [Fe(CN)_6_]^4−/3−^ added in solution, as result of antigen-antibody binding. Selectivity of the detection was proven with proteins typically found in food matrices such as ovalbumin, casein, lysozyme and bovine serum albumin [40]. Reproducibility was very good considering the general variability of screen-printed electrodes and manual modifications steps, with RSD of 1.6%–6.0% (n = 3) over the concentration range from 1 pg·mL^−1^ to 100 ng·mL^−1^. Although the authors did not include a direct comparison with sensors lacking graphene, the good sensitivity reflected by the detection limit of 0.85 pg·mL^−1^ was attributed in part to the excellent conductivity of graphene, promoting the charge transfer of [Fe(CN)_6_]^4−/3−^ probe [40]. Efficient functionalization and high density immobilisation of antibody were other contributing factors to high analytical performances of the sensor that were comparable or better than those of more complex sensors relying on amplification steps. One promising feature of this immunosensor with regards to practical applications, besides its simplicity, was the successful analysis of β-lactoglobulin in cake, biscuit and snack samples, in good agreement with results of ELISA analysis conducted in parallel [40].

#### 4.1.2. Biosensors for Egg Allergens

Egg allergies affect mostly children under 3 years old. The major allergens from egg white—ovalbumin (which represents 54% of egg white proteins), ovotransferrin (12%), ovomucoid (11%), ovomucin (3.5%), and lysozyme (3.5%)—have however many potential applications in food processing or as nutraceutical and pharmaceutical agents [102]. Despite good manufacturing practices implemented in industry and labelling rules imposing clear specification of eggs or egg proteins present in the composition of food products, the potential for accidental contamination remains (e.g., by inadequate cleaning of production lines). An appropriate analytical control off egg proteins is very important to ensure the safety and quality of products and as the most abundant protein in egg white, ovalbumin can be used as a marker to assess the contamination of food products with eggs. Ovalbumin is a 42.7 kDa glycoprotein with an isoelectric point of 4.5, one of the very first proteins to be studied. There were several biosensors developed for ovalbumin, all based on antibodies as no specific aptamer has been selected until now. Recent bioanalytical methods include one magnetoassay and one immunosensor relying on screen-printed electrodes (Table 1). To construct the immunosensor, the authors functionalized the surface of a screen-printed carbon electrode with carboxyphenyl diazonium salt generated in-situ and used the carboxyl groups to anchor covalently the anti-ovalbumin antibody by amine coupling [41]. The peak current for [Fe(CN)_6_]^3−/4−^ recorded by DPV was linearly correlated with the logarithm of ovalbumin concentration in the range 1 pg·mL^−1^ to 0.5 μg·mL^−1^ and a detection limit of 0.83 pg·mL^−1^ (S/N = 3) was reported. Biosensor selectivity was demonstrated through the negligible responses recorded when testing four non-specific food proteins; β-lactoglobulin, BSA, egg lysozyme and casein. Method accuracy was investigated by spiking egg-free cake sample extracts with ovalbumin at three concentration levels, the obtained recoveries being 104%–109.6%. This is one of the very few studies on allergen electrochemical biosensing reporting on the storage stability of the sensor: less than 2% change in response was recorded for 10 ng·mL^−1^ OVA after 14-days at 4 °C [41]. 

**Figure 7:**
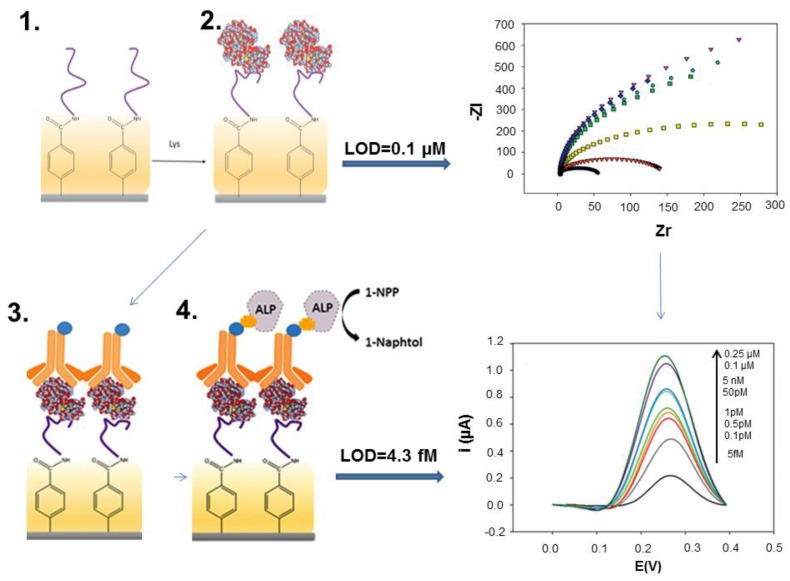
Illustration of two configurations used in screen-printed aptasensors for lysozyme analysis in wine. Steps 1 and 2 depict the construction of an impedimetric aptasensor where a biotinylated aptamer is covalently immobilized on a carboxyl-functionalized surface (**1**). Lysozyme binding (**2**) is detected directly by EIS. In a second assay, a biotinylated antibody is added after lysozyme binding (**3**), this detection antibody is labeled with streptavidin-alkaline phosphatase conjugate (ALP), the enzyme substrate, 1-naphtyl phosphate (1-NPP) is added (**4**) and the enzymatic product 1-naphtol is detected by DPV. Introduction of steps 3 and 4 and detection by DPV allowed improving the detection limit from 0.1 µM to 4.3 fM. Adapted from [39] and [34] with permission.

Lysozyme, also called “muramidase” is a 14 kDa enzyme found in the human body, bird eggs, plants and bacteria that catalyses the hydrolysis of glycosidic bonds in peptigoglycan, in the cellular walls of Gram-positive bacteria [99]. Highly relevant to human health as “body’s own antibiotic”, lysozyme has also significant economic importance in the food industry–particularly in the production of wine, beer, cheese and sausages- as a fining or antimicrobial agent [99]. The maximum amount allowed for use in wines as per the International Organization of Wine is 500 mg L^−1^. Since the development of the first lysozyme aptamer by Cox and Ellington by SELEX in 2001 [53] lysozyme was one of preferred target analytes in the development of new biosensors based on various configurations. A second aptamer was selected in 2010 by a different method, capillary electrophoresis-SELEX (CE-SELEX) [56] and started to be applied in biosensors as well. Aptamer-based electrochemical detection of lysozyme was recently reviewed [99] showing many possible biosensing strategies that range from direct detection to more complex assay formats involving two probes, e.g., “sandwich” between an aptamer and an antibody, three-way junction DNA constructs etc. However, only few studies have included analysis of real food samples such as chicken egg and wine and were performed with SPEs (Table 1). The advantages and limitations of a more complex versus a simple sensing configuration can be illustrated by two biosensors developed by some of us for wine analysis. In a first study, the two lysozyme aptamers, selected by different procedures were compared as biorecognition elements in impedimetric screen-printed biosensors based on screen-printed carbon electrodes. The electrode surface was modified via diazonium chemistry to introduce carboxyl group, to which amine-ended aptamers were covalently attached by amine coupling. The analytical performances of impedimetric biosensors based on the two aptamers can be judged as similar [39]. In a follow-up study, a more complex configuration was used where lysozyme was sandwiched between the aptamer affixed on electrode surface as previously and a detection antibody [34]. The antibody was enzymatically labeled with ALP, the enzyme substrate was added and the enzyme product, 1-naphtol was detected by DPV. The concentration of lysozyme in the samples was directly related to the intensity of current due to the oxidation of 1-naphtol by DPV. With this biosensor, it was possible to reach a detection limit of 4.3 fM versus 0.1 µM achieved previously with the impedimetric biosensor (Figure 7). The linear range was wide, from 5 fM to 5 nM and the reproducibility was good, although the biosensor construction involved several steps, all performed manually.

Residual amounts of lysozyme in wine being quite small, particularly in bentonite-treated wines [2], a high sensitivity of the biosensor is required. On the other hand in chicken egg the lysozyme amount is much higher and a simpler, less sensitive biosensor would be appropriate. For any application, one should balance the requirements for sensitivity with the complexity of the analytical method.

Besides aptamers, a 2006 study [46] showed the possibility to detect 1 µg·mL^−1^ lysozyme in buffer solutions using a MIP formed by electropolymerisation at the surface of a SPPtE (Table 1). Surprisingly, no research has followed on lysozyme detection in food using MIP-modified SPEs, yet MIPs for lysozyme detection based on other methods are actively researched [103].

#### 4.1.3. Biosensors for Gluten

**Figure 8:**
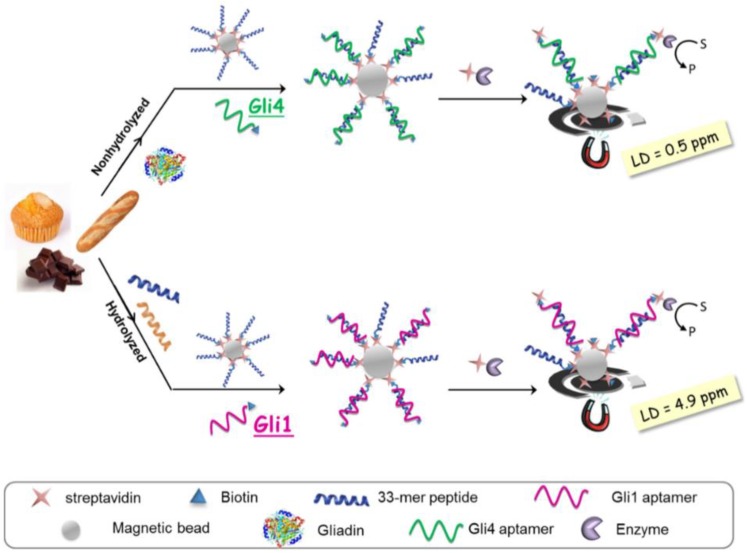
Principle of aptamer-based magnetoassays for gliadin determination in processed foods with electrochemical detection. Reprinted from [104] under the terms and conditions of the Creative Commons Attribution (CC-BY) license (http://creativecommons.org/licenses/by/4.0/).

“Gluten” designates a mixture of proteins from wheat, barley and rye that elicit severe reactions in people with gluten intolerance or suffering from celiac disease. These people need to respect a strict, lifelong diet that is either low-gluten or gluten-free (for people affected by celiac disease). Gluten is composed of two main groups of proteins classified on the basis of their different solubility in alcohol-water mixtures: prolamins (soluble) and glutenins (the insoluble fraction). A rough estimation currently used to calculate gluten considers a 1:1 ratio between prolamins and glutenins. Prolamins from wheat, barley, rye and oats are called gliadins, hordeins, secalins and avenins, respectively. As per current legislation, gluten-free food is defined as containing less than 20 ppm gluten. Increased consumer awareness, strict labeling rules in place today and the huge market of gluten-free products prompted an increased analytical testing of gluten and drove the research towards more sensitive, faster and cost-effective detection methods. Despite sustained research efforts over the last 20 years, gluten detection remains a very challenging analytical problem [104] due to: (i) the need for quantitative extraction of toxic protein and peptide fragments from various types of food matrices, either unprocessed or heated, fermented and hydrolysed; (ii) availability of standards and reference materials and (iii) selectivity and sensitivity of detection. In particular, the method should not show cross-reactivity to non-toxic proteins from other cereals (e.g., soy, maize or rice). Commercial ELISA kits are largely used for sensitive gluten analysis based on several monoclonal antibodies raised against various repetitive toxic peptide fragments from gluten. The limit of detection achieved with ELISA kits range from 0.6 ppm to 10 ppm gluten depending on the assay format (sandwich, competitive or lateral flow device) and antibody [104]. Recent breakthroughs in gluten analysis include the development in 2014 of aptamers for gluten by two independent research groups and the launch of the NIMA sensor (www.nimasensor.com), available commercially in fall 2016. NIMA is a portable device for personal use, able to analyse portions of food and providing responses in 2 min, if the food is gluten-free or not (i.e., containing less than 20 ppm gluten or over this level). One of the 25 best innovations of 2015, NIMA uses spectroscopy and specifically detects gluten, however is not suitable for fermented and hydrolysed foods. In hydrolysed foods, gliadin is broken in small peptides for which antibodies and aptamers developed for the native form of gliadin don’t have enough affinity. One response for a sensitive, selective and accurate analysis of gliadin in all types of food matrices, both unprocessed and fermented/hydrolysed could rely on using a mixture of aptamer sequences, rather than a single one. A competitive electrochemical aptamer-based magnetoassay was described based on screen-printed electrodes and two aptamer sequences selected against the immunodominant peptide from gliadin, the 33-mer [104]. The most abundant clone in the selection pool during the aptamer selection process, Gli1 with a dissociation constant K_D_ = 101 ± 11 nM determined by chronoamperometry, was found suitable for quantifying gluten in hydrolysed samples, reaching a detection limit of 4.9 ppm. The other second sequence used, Gli4 was the one with the highest affinity among the selected aptamer sequences (K_D_ = 61 ± 4 nM by chronoamperometry) and enabled achieving a detection limit of 0.5 ppm and quantification of gluten in heated foods. The aptasensing method involved functionalisation of magnetic beads with 33-mer peptide (Figure 8).

Next, sample extracts containing the toxic gliadin fragments were mixed with the magnetic beads and with a biotinylated aptamer (either Gli 1 or Gli4, depending on the type of sample). Gliadin peptides in the sample competed with the 33-mer peptide on the MBs for binding with the aptamer. The MBs were concentrated on electrode surface by using a magnet. Gluten detection was accomplished by labelling the aptamer via a streptavidin-enzyme conjugate and conversion of enzymatic substrate into an electrochemically active product that is finally determined at electrode surface. Remarkably, the authors went further towards practical applications by comparing the aptassay with current official ELISA method, finding a good agreement [104].

A genosensor for the detection of DNA encoding a repetitive gliadin fragment was also reported. Martín-Fernández et al. [97] described a classic sensor in a “sandwich” configuration where the target DNA was hybridized with both the capture probe affixed to the electrode substrate and with a signalling probe. The signalling probe was contiguous with the capture probe and in order to achieve detection of target DNA the signalling probe was labeled with an enzyme (HRP) via streptavidin-biotin affinity interaction. The enzymatic substrate, TMB was then added and the reaction product was detected by chronoamperometry. The study [97] emphasized the importance of the capture probe conformation to achieve a selective detection. While a linear capture probe enabled a lower limit of the linear range compared to a hairpin probe (1 nM versus 5 nM) the selectivity was much better when a 9-bp shared-stem haipin probe was used and allowed to detect DNA encoding gliadin from wheat without response to DNA from rye and barley.

#### 4.1.4. Biosensors for Peanut Protein Allergens

The high prevalence of peanut allergy compared to other food allergies, particularly in western countries and the seriousness of allergic reactions, going up to breathing and heart problems and even anaphylactic shock and death raise serious concerns for consumers and food industry with regards of preventing incidents related to food consumption by allergic individuals and control of peanut-containing products. Seventeen proteins, named Ara h 1 to Ara h 17 have been identified so far in direct relation with peanuts’ allergenic potential, according to the allergen nomenclature [105] posted by WHO/IUIS Allergen Nomenclature Sub-Committee. From these, Ara h 1, Ara h 2 and Ara h 3 were indicated by earlier studies as the major allergens while some recent studies point to Ara h 2 and Ara h 6 as posing the greatest risk to sensitized individuals [106]. Ara h 3 is the most abundant protein in peanut extracts, followed by Ara h 1, Ara h 2 and Ara h 6 [107]. Ara h 1 is a homotrimer with a MW of 150 kDa [107] (each monomeric unit having 64 kDa [105]). Ara h 3 is a hexamer with a MW of 360–380 kDa [107] and the other peanut allergens are monomers with MW of 8–17 kDa [105]. Ara h 1, Ara h 2, Ara h 3 and Ara h 6 have isoelectric points around 5. Ara h 1 and Ara h 3 belong to the cupin protein superfamily, while Ara h 2 and Ara h 6 are conglutins [105]. 

Many efforts were directed towards development of biosensors as fast, sensitive and cost-effective alternative methods for peanut allergens. Most biosensors were based on antibodies, however an aptamer specific for Ara h 1 has been developed in 2013 by capillary electrophoresis (CE)-SELEX [71] having an affinity constant Kd of 353 ± 82 nM (determined by surface plasmon resonance) and no response towards other proteins, including Ara h 2. The aptamer was soon used as biorecognition element in biosensors, including electrochemical ones. Electrochemical screen-printed biosensors for peanut allergens have been developed so far for Ara h 1 [12], Ara h 2 and Ara h 6 (Table 1). A magnetoimmunosensor for the dual detection of Ara h 1 and Ara h 2 was reported by Ruiz-Montiel et al. [90]. Detection of Ara h 1 and Ara h 2 in different food extracts such as wheat flour hazelnuts, peanuts, chocolate bars with roasted peanuts and peanut cream and in spiked wheat flour samples was also described as an application. The results obtained with the dual amperometric immunoassay agree to those obtained by ELISA and the assay was reproducible, as relative standard deviation (RSD) values of 7.3% and 8.9% were calculated for Ara h 1 and Ara h 2 (n = 8 electrodes). Evaluation of the selectivity of the dual magnetoimmunosensor consisted in measuring the response for 0 and 500 ng·mL^−1^ Ara h 1 and 0 and 1.0 ng·mL^−1^ Ara h 2 in the absence and in the presence of BSA, lysozyme and OVA, proteins which can coexist with the peanut allergens in food extracts. No interferences and no cross-reactivity between Ara h 1 and Ara h 2 were observed in these conditions. The authors further described a procedure for total detection of Ara h 1 and Ara h 2 at a single SPCE, relying on mixing magnetic beads modified with Ara h 1 and Ara h 2 antibodies and depositing them on a single electrode. Such sensor would be useful for checking, for example food contamination with peanuts.

Lopez et al. [65] described an electrochemical genosensor for the detection of an 86-mer DNA peanut sequence encoding part of Ara h 2. The biosensor was based on a sandwich format and allowed to achieve a sensitivity of 3 µA·nM^−1^ and a detection limit of 10 pM DNA. 

Ara h 2 and Ara h 6 have similar allergenic activity, share 59% of amino acid sequence, and both are thermally stable and resistant to proteas·e enzymes in the digestive tract. A voltammetric immunosensor for Ara h 6 was recently developed [100] based on a screen-printed carbon electrode modified with Au NPs and a sandwich-type assay using two-monoclonal mouse IgG antibodies against Ara h 6. To translate allergen binding into an electrochemical signal, the detection antibody was labeled with alkalinephosphatase (ALP) via avidin-biotin affinity and 3-indoxylphosphate (3-IP) and silver nitrate were added in the reaction medium. The detection of Ara h 6 was based on enzyme-catalyzed silver precipitation and electrochemical oxidation of deposited silver by linear sweep voltammetry in the range from −0.02 to + 0.4 V. The immunsensor was validated and applied for the detection of Ara h 6 in cookies and chocolate samples. Accuracy was estimated by analysing cookies intentionally spiked with 5, 10 and 50 ng·mL^−1^ Ara h 6 and the recoveries ranged between 96.7% and 98.8%.

#### 4.1.5. Biosensors for Hazelnut Allergens

Hazelnuts (*Corylus avellana*) account for an important segment of the world’s trade of tree nuts after almonds, pistachios, walnuts and cashews, with the global production amounting to 337, 870 metric tons and a supply value of $3717 million in 2014 [108]. While used for many applications in the food industry, hazelnuts have also a high allergenic potential for sensitized people, which react to either the nut itself, the pollen of the hazelnut tree or to both. A comprehensive discussion on hazelnut allergens and biosensing strategies developed so far for their detection was recently published [7]. As summarized, there are 10 groups of allergenic “corylin” proteins characterised so far in hazelnuts, namely: Cor a 1, Cor a 2, Cor a 8, Cor a 9, Cor a 10, Cor a 11, Cor a 12, Cor a 13, Cor a 14 and Cor a TLP. Some of these groups include various isoallergens and each isoallergen might present different isoforms. Various ELISA tests have been described in literature for hazelnuts detection in cookies, biscuits, chocolates, breakfast cereals and cereal bars, ice-cream, olive oil, etc., with LOD of 0.1–3 mg/kg, as summarized by Alves et al. [7]. To these add mass-spectrometry-based methods and methods based on detection of DNA encoding the allergen proteins (real-time PCR, etc.). A few biosensors have also been developed, mostly optical ones. There is only one example of electrochemical screen-printed biosensors so far, namely a DNA array for PCR amplified detection of hazelnut allergens in foodstuffs [64]. The DNA array consists of eight screen-printed Au electrodes modified with thiolated DNA capture probes and blocked with 6-mercapto-1-hexanol. The target analytes were fragments of the genes related to the expression of Cor a 1 isoallergen proteins Cor a 1.03 and Cor a 1.04. The DNA was extracted from food samples with a commercial extraction kit and was amplified by PCR. The capture probes were DNA sequences, complementary to the target DNA. A sandwich configuration was used where the target DNA fragments were hybridized with capture probes at the surface of the sensor and with signaling probes, labeled with alkaline phosphatase (ALP). The detection of target DNA was enabled via enzymatic transformation of ALP substrate 1-naphtyl phosphate and electrochemical detection of the enzymatic product, 1-naphtol by DPV (Figure 2). Although the construction of such genosensors requires optimisation of several steps, a very important advantage is that this “generic” sensor design could be used for simultaneous detection of multiple target analytes (e.g., multiple allergens), simply by adequately changing the capture and signalling probes. 

The genosensor array for hazelnut allergens allowed detecting hazelnut amplicons in the linear range of 1–20 nM. The cross-reactivity between individual sensors was non-significant and the non-specific signal was very small. Importantly, the genosensor compared well with an ELISA kit and revealed the presence of hazelnuts in various types of food including hazelnuts, chocolate, soymilk, biscuits, breakfast cereal, snacks and lecithin supplements. Food products such as ketchup, peanuts and peanut butter were also investigated as negative controls. Additionally, the results obtained with the electrochemical compared well to the elecrophoretical analysis of Cor a 1.03 and Cor a 1.04 amplicons.

### 4.2. Figures of Merit of SPE-Based Biosensors for Allergens

The analytical performances of screen-printed biosensors for allergens such as limit of detection, linear range and selectivity were adequate for analysis of real samples as illustrated by data in Table 1. The time per assay was generally between 1 h and 2 h, as many methods required multiple incubation steps, however it reached almost 4 h for one sensor [94]. Although in general the test duration with screen-printed biosensors was significantly lower than ELISA, it remains rather long for practical applications and sample preparation is also a bottleneck. Future biosensor developers need to put in balance the need for high sensitivity (requiring longer incubation or addition of amplification steps which make the assay longer) with the one for fast analysis, bearing in mind recent development such as the Nima sensor for gluten able to provide a response in 2 min. The biosensors for allergen detection were conceived as disposable devices and their reproducibility was rather good considering that were several modification steps performed manually. The storage stability of the biosensors was little investigated and this area will definitely benefit from further research. For a genosensor for gliadin stored at 4 °C in humid atmosphere, Martin-Fernandez et al. [97] found a response decrease to 74.1% (after 2 days) and to 72.8%, (after 10 days). Sensor selectivity was generally proven by challenging the biosensors with non-specific proteins that might be found in food matrices. The applications summarised in Table 1 underline the good selectivity of the biosensors for allergens.

### 4.3. Challenges Associated with Real Samples Analysis 

The examples of electrochemical biosensors applied so far for the detection of allergens in wine, egg white, chocolate, biscuits, snacks, cakes, etc. (Table 1) prove the potential of these bioanalytical devices for the analysis of real samples. Electrochemical interferences were eliminated by a combination of well-protected interfaces against non-specific adsorption, adequate analysis protocols including washing steps and sample preparation, including typically a high dilution factor. Non-specific binding of compounds present in the sample matrix to the transducer surface resulting in a change of electrical properties at the interface and potential interference with biosensor response was prevented by blocking with small thiols such as mercaptohexanol (Au electrodes and electrodes with Au NPs), bovine serum albumin, whole milk and casein. The analysis protocols typically involved transferring the biosensor after incubation with the sample into a solution containing the electrochemical reporter probe, thus avoiding interferences from electrochemically active compounds that might be present in the sample solution. Several studies reporting on new biosensors for allergens estimated the accuracy and suitability of the method for real samples by analyzing intentionally spiked “allergen-free” food. Very good recovery factors in the range 95%–110% were found for samples spiked at three concentration levels. It is worth mentioning that the spiking should be performed at the very beginning, before applying any sample treatment, cleaning or dilution procedure, otherwise the effectiveness of sample preparation and effect of sample preparation on allergen-bioreceptor affinity remains unknown. Other biosensor studies included comparison with current standard ELISA [90,96] and a few even described detailed method validation [100]. Such studies could serve as models to follow for future research to enable the progress towards practical applications of biosensors for allergens.

Besides the above-mentioned problems generally encountered in the development of biosensors, allergen analysis faces a number of additional challenges. These are related to sample preparation, changes suffered by allergen proteins during food processing, availability of standards and reference materials and lack of established maximum allowed levels for each allergen. In some cases, the sensitivity that will be required for a newly developed method can be derived from current labeling rules. For example in Europe, milk and egg proteins should be indicated on wine label if they were used for fining and were detected by an analytical method with detection and quantification limits of 0.25 and 0.5 ppm respectively [109]. In Japan, seven allergens must be indicated on product labels if they are present at more than 10 ppm [3]. As per Codex Alimentarius, “gluten-free” food is defined as having under 20 ppm gluten. The case of gluten provides a nice illustration of the complexity of problems associated with sample preparation, the changes suffered by allergen proteins during food processing and availability of standards and reference materials [60,104,110,111]. Sample preparation for allergen analysis with biosensors generally follows the well-established protocols for ELISA, consisting in extracting the sample with an appropriate buffer while heating, followed by centrifugation and dilution. In samples where the allergen protein is bound to phenolic compounds (chocolate, wine) this interaction must be broken first. However there is a significant variability between results provided by laboratories performing ELISA with different extraction cocktails and protocols and sample preparation remains an area where improvement will undoubtedly follow in the future. For biosensors, the sample preparation procedure, while ensuring quantitative extraction of allergen from the food matrix, should not cause changes in protein structure that will affect the binding to its bioreceptor. Aptamer affinity for the target analyte is drastically influenced by ionic strength and nature of ions in the sample solution [55]. One approach solving both the problem of electrochemical interferences and ensuring the preservation of aptamer affinity as displayed with standard allergen solutions is to use a high enough dilution factor. This procedure, afforded with sensitive biosensors, could introduce however errors and should be used with caution. Food processing involving heating, fermentation and hydrolysis could lead to major changes in protein structure and extensive fragmentation. Antibodies developed for the native gliadin for example show little affinity to deamidated gliadin and to small peptide fragments present in hydrolysed products and appropriate bioreceptors should be considered for analysis of processed food. Additionally, small peptide fragments are not suitable for sandwich formats as these require accommodating two epitopes-another aspect to consider in biosensor development. The lack of commercially available standard proteins and reference materials for some allergens makes very difficult the estimation of accuracy of new bioanalytical methods-including biosensors- and the comparison with other studies by different laboratories. For gluten, it is approximated that gliadin represents half of the total amount of gluten. Gliadin from Sigma (Taufkirchen, Germany), PWG standard (obtained by processing 28 wheat cultivars) [112] and different gliadin peptides were all used as standards in the bioanalytical determination of gluten. All these aspects should be considered before developing a new biosensor for allergens.

### 4.4. Current Trends in Allergen Analysis with SPE-Based Biosensors

Recent trends in the development of electrochemical biosensors for allergens based on screen-printed electrodes mirror the general tendencies manifested with biosensors, including:

(1) Use of nanomaterials such as Au NPs, graphene or carbon nanotubes to increase the effective area, improve conductivity and facilitate the quantitative immobilization of bioreceptors [98].
(2) Development of small portable biosensors controlled by smartphones
(3) Multi-analyte sensors and arrays

In a given food matrix, several allergens may co-occur (e.g., milk, almonds, hazelnuts, peanuts in chocolate or gluten, milk, peanuts and tree-nuts in cakes, snacks or biscuits etc.) and their simultaneous analysis using multi-analyte arrays would be advantageous. In genosensors also targeting multiple genes encoding various allergens would be beneficial. So far, only a dual biosensor was reported for analyzing gluten in non-hydrolyzed and hydrolyzed food, using two aptamers, each one immobilized on a different electrode. Simultaneous quantitative detection of multiple targets does have some challenges related to the density of immobilized probes and optimization of operational conditions, however this is an area that will undoubtedly see development in the future, potentially by adapting some concepts developed for biomedical field.

(4) Amplification of analytical signal to achieve high sensitivity of detection.

Enzymatic amplification was a preferred way to achieve sensitive detection of allergens. New schemes for signal enhancement based on nanomaterials, carefully designed DNA sequences and aptamers as those used for other analytes relevant for food quality and safety [11] could be envisaged in the future.

(5) Development and use of better biorecognition receptors with improved stability and affinity.

Currently, aptamers are only available for a small number of allergens such as lysozyme [53,56], Ara h 1 [71], gliadin [59,113], and β-conglutin (lupin) [58], however the race is on for developing aptamers for other allergens. With the advances in aptamer selection technology the following years will witness the selection of an increased number of aptamers with high affinity and selectivity. Still, as aptamer development can be quite challenging, it is anticipated that antibodies will continue to be used in allergen biosensing in the coming years. Besides aptamers, novel bioreceptors such as nanobodies, peptide nucleic acids etc as well as “artificial antibodies”—MIPs are continuously being developed for a variety of molecules (see for example the recent review on toxin biosensing [114] or MIPs applications in protein detection [66] and food safety [115]). Lysozyme binding by camelid single domain antibody was previously explored [116] targeting lysozyme–expressing tumors [117], however the new bioreceptor could be also applied in the future in allergen biosensing in food. Increased interest in tapping the potential of MIPs for fast detection of food allergens, demonstrated among others by patents on peanut allergens [71] is promising also for development of new concepts based on screen-printed electrodes, in addition to already developed optical tests.

## 5. Conclusions and Perspectives 

Recent years have witnessed the emergence of electrochemical biosensors for allergen analysis based on screen-printed electrodes. Although the examples are not numerous, the various analytical strategies developed until now lay the foundation for more sensors with even better analytical performances. The biosensors for allergens will benefit from progress in both bioreceptor development (as selection of new aptamers against allergens is intensively researched) and advances in screen-printed electrodes as new interfaces with better conductivity and loading capacity with biomolecules are continuously developed, including using new nanomaterials. New amplification schemes are increasingly reported. More interlaboratory studies will contribute to establishment of standard and reference materials and improvement of sample preparation for fast and effective allergen extraction. Screen-printed biosensors have a huge potential to provide highly sensitive, cost-efficient, disposable analytical tools, and many of biosensor modification steps, currently performed manually, are compatible with mass-production techniques. To approach their potential and reach commercial success, biosensors for allergens based on screen-printed electrodes need to include new biosensing strategies that reduce the analysis time while continuous focus should be placed on comparative studies with current standard methods implemented in food testing laboratories. 

## Figures and Tables

**Table 1:** Examples of biosensors for the detection of allergens based on screen-printed electrodes.

Allergen/Real Matrix	Biorecognition Details	Detection Method and Electrode Details	Analytical Characteristics	Reference
β-casein	Direct detection; Protein A/Ab	SPCE pretreated EC; direct oxidation of casein by DPV	Detection of 1, 10 and 100 µg·mL^−1^	[36]
β-casein	Competitive; β-casein/Ab/HRP-labeled anti-IgG Ab	SPCE-plasma activated; amperometric reduction of BQ at −0.28 V;	LR: 0–10 ppm	[35]
Bovine casein and bovine gamma globulin G (bIgG) in milk	Direct; QD-labeled Ab on screen-printed foldable membrane	Bi-SPCE with bismuth citrate; ASV of Pb and Cd from QD s	DL: 40 ng·mL^−1^ (casein)	[91]
			DL 20 ng mL^−1^ (bIgG)	
β-lactoglobulin/cake, cheese snacks, sweet biscuits	Direct; Ab/β-lactoglobulin	GR-SPCE; DPV of [Fe(CN)_6_]^3−/4−^	LR: 1 pg·mL^−1^ to 100 ng·mL^−1^	[40]
			DL:0.85 pg·mL^−1^	
β-lactoglobulin/milk	Sandwich; magneto immunoassay; Ab-MBs/β-lactoglobulin/ HRP-Ab	SPCE; amperometry, reduction of BQ at −0.2 V	LR: 2.8–100 ng·mL^−1^	[89]
			DL: 0.8 ng·mL^−1^	
Ovalbumin	Sandwich; magneto immunoassay; Ab-MBs/ovalbumin/HRP-Ab	SPPtE; LSV of H_2_O_2_ using thionine as mediator	LR:11–222 nM	[82]
			DL: 5 nM	
Ovalbumin/spiked cake extract	Direct; Ab/ovalbumin	GR-SPCE, DPV of Fe(CN)_6_]^3−/4−^;	LR: 1 pg·mL^−1^–0.5 μg·mL^−1^;	[41]
			DL: 0.83 pg·mL^−1^	
Genes encoding hazelnut allergens Cor a 1.04 and Cor a 1.03)/hazelnut, chocolate, soymilk, biscuits, lecithin supplements, ketchup, snack, breakfast cereal, peanut butter	Sandwich; tCP/PCR products/bSP/SA-ALP	SPAuE;DPV of α-naphthol	LR: up to 20 nM [Cor a 1.04, Cor a 1.03]	[64]
			DL: 0.3 nM (Cor a 1.03) 0.1 nM (Cor a 1.04)	
Gene encoding Ara h 2 peanut protein	Sandwich; tCP/target DNA + bSP/SA-ALP	SPAuE; DPV of 1-naphtophenol	LR: 50 pM–50 nM	[65]
			DL: 10 pM;	
			RSD: 7.22% (n = 8)	
			S: 3 µA/nM	
Ara h 1/food extracts (hazelnuts; peanuts, chocolate bars; chocolate chip cookies; peanut creams; peanut oil) and saliva	Sandwich; cAb-MB/Ara h 1/bAb/SA-HRP	SPCE, amperometry, HQ detection at −0.2 V	LR: 20.8–1000 ng·mL^−1^	[96]
			DL: 6.3 ng·mL^−1^	
Ara h 1/cookies and chocolate	Sandwich; cAb/Ara h 1/b-Ab/SA-ALP	AuNPs-modified SPCE; LSW for stripping enzymatically deposited Ag	LR: 12.6–2000 ng mL^−1^	[94]
			DL: 3.8 ng·mL^−1^	
dual detection of Ara h 1 and Ara h 2/ wheat flour, hazelnuts; peanuts; chocolate bars with roasted peanuts and peanut cream;	Sandwich; Magneto-immunoassay; cAb-MBs/Ara h 1/b-Ab/SA-HRP-polymer (Ara h 1); cAb-MBs/Ara h 2/Ab/F(ab’)_2_-HRP (Ara h 2)	Dual SPCE; amperometry, HQ detection at −0.2 V	Ara h 1:	[90]
			LR: 60–1000 ng·mL^−1^	
			DL: 18 ng·mL^−1^	
			Ara h 2:	
			LR: 0.25–5 ng·mL^−1^	
			DL: 0.07 ng·mL^−1^;	
Ara h 6/chocolate and cookies	Sandwich; cAb/Ara h 1/b-Ab/SA-ALP	AuNPs-modified SPCE; LSW of enzymatically deposited Ag	LR: 1–100 ng·mL^−1^	[100]
			DL: 0.27 ng·mL^−1^;	
			RSD ≤ 9.8% (n = 6)	
Gluten/gluten-free foods (including heat-treated and hydrolysed)	Competitive; 33-mer peptide-modified MBs/b-APT+gliadin/SA-HRP. APT: Gli 1 or Gli 4	SPCE; chrono-amperometry at 0 V of TMBox	DL: 4.9 ng·mL^−1^ (Gli 1);	[61]
			DL: 0.5 ng·mL^−1^ (Gli 4)	
Sequence encoding the 33-mer peptide in wheat/wheat flour	Sandwich; tCP/target DNA + FITC-SP/anti-FITC-Fab–HRP	SPAuE; chronoamperometry at −0.2 V of TMB_ox_ for 60 s	LR: up to 50 nM;	[66]
			DL: 0.3 nM	
DNA encoding gliadin fragment	Sandwich; tCP/target DNA + b-SP/SA-HRP	SPAuE; chrono- amperometry at 0 V of TMB_ox_	LR: 5–50 nM	[97]
			DL: 1 nM	
			RSD (n = 3) = 21% (10 nM) and 4.3% (5 nM).	
Lysozyme/wine	Direct; APT Cox/lysozyme and APT Tran/lysozyme	SPCE/EIS	Apt Cox:	[39]
			LR: 0.1–0.8 μM	
			DL: 100 nM	
			Apt Tran:	
			LR: 0.025–0.8 μM	
			DL:25 nM	
Lysozyme/wine	Sandwich; APT/lysozyme/ALP-Ab	SPCE; DPV, 1-naphtol	LR: 5 fM– 5 nM	[34]
			DL: 4.3 fM	
Lysozyme/egg white	Direct; APT/lysozyme	AuNP-SPCE; SWV, [Ru(NH3)_6_]^3+^	LR:1–50.0 pg·mL^−1^	[43]
			DL: 0.3 pg·mL^−1^	
Lysozyme	Direct; APT/lysozyme	MWCNT–SPCE, EIS [Fe(CN)_6_]^3−/4−^	DL: 12.09 ng mL^−1^ (862 nM)	[42]
Lysozyme	Direct; MIP/lysozyme	SPPtE, CV	1 µg·mL^−1^ leads to 30.3% reduction in current, compared to 4.5% for control	[46]

Abbreviations: Ab-MBs: magnetic beads modified with capture Ab; tCP: thiolated capture probe; bSP: biotinylated signalling probe; SA-ALP: streptavidin–alkaline phosphatase conjugate; GR-graphene; SPCE, SPAuE, SPPtE: screen-printed carbon, gold and platinum electrode; LSV: linear sweep voltammetry; b-APT: biotinylated aptamer; TMB_ox_: oxidized TMB formed in the reaction with H_2_O_2_ catalysed by HRP; FITC-SP: FITC-labeled signalling probe. Au NPs: gold nanoparticles; MWCNT: multiwall carbon nanotubes. Bi-SPCE SPCE loaded with bismuth citrate; ASV: adsorbtive stripping voltammetry.
